# How to Secure Good Dental Organs, and Preserve Them from Decay, Prevent Rickets, Hip Disease, Etc.

**Published:** 1884-07-20

**Authors:** H. E. Dennett

**Affiliations:** Boston


### HOW TO SECURE GOOD DENTAL ORGANS AND PRESERVE THEM FROM DECAY, PREVENT RICKETS, HIP DISEASE, ETC.

By H. E. DENNETT, D.D.S., BOSTON.

It is conceded that dental decay is the dissolving away of lime salts by vitiated secretions. This is not due so much to a want of cleanliness of the mouth as is generally supposed. It is not true that "A clean tooth never decays." One may devote twelve hours out of the twenty-four to the ablution of the mouth and fail to prevent decay of the teeth, so long as Nature's dietic laws are violated. Acid will dissolve lime whenever the two meet. Acid saliva may be expected to follow an excessive use of acids, or of those elements which are capable of being converted into acids, or from a deficiency of the opposite elements.

Perfect health includes a perfect set of teeth. The teeth are little indicators that denote by their condition that of the whole system, just as a thermometer indicates thermal changes.

Stripped of all mystery, the rule for health so far as food is concerned is simplicity itself. Nature has given to every one an appetite which, in its normal condition, may be relied upon to make a proper choice of foods. Select, then, the food for which the appetite calls, containing all its natural elements, and Nature will take care of the results. Dental development in man is discernable as early as the seventh week of intra-uterine life, hence the importance of a strictly correct diet from the first, if mothers desire to give birth to children who may have perfectly formed teeth. The lime from her teeth will be dissolved, taken into the circulation and appropriated by the offspring. As a consequence, the mother who passes through the periods of gestation and lactation without a sufficient amount of bone and tooth element in her food will suffer from loss of teeth, neuralgia, rheumatism, and other diseases which result from an impoverished state of a system drained to its utmost. Excepting civilized man, all flesh-eating animals take as much of the bone with the flesh they consume as they can break with their teeth sufficiently fine to swallow, and all have good dental organs. Take from any carnivorous animals their supply of bone which Nature furnishes with the flesh and dental decay will be the inevitable result. Several years ago the lions in the Zoological Gardens of London were fed upon the thighs of horses which were too large for them to break and eat. As a consequence their young were born with cleft palates and died. Subsequently they were fed upon deer and other small-boned animals, and their young were born with perfectly formed palates and lived. Veterinary surgeons have long known that certain diseases of their dumb patients can only be successfully treated by feeding them with bone meal. A dam too aristocratic to gnaw bones gave birth to successive litters of rickety pups; but after eating food which contained a liberal supply of bone meal, she produced perfectly healthy ones, and by the same sire. Arguments in favor of eating bone to prevent the decay of the teeth, as well as to cure a long catalogue of bone and kindred diseases might be continued indefinitely; but as "A word to the wise is sufficient," it seems only necessary to add that a long and continued experiment has been made upon a family with most satisfactory results. The bones used were selected from perfectly healthy animals, none being accepted that bore the slightest blemish, carefully cured without being allowed to pass through any perceptible chemical changes, finely granulated and incorporated into soups, gravies, bread, etc., in the proportion of from one to three spoonfuls to each pint of gravy, soup or flour. The relative proportion of nutritive elements in one hundred parts of different kinds of animal food have been found as follows: beef 26, mutton 29, pork 24, chicken 27, milk 7, bone 51.—*Amer. Jour. of Pharmacy.*

